# Prevalence of diabetes and hypertension and association with various risk factors among different Muslim populations of Manipur, India

**DOI:** 10.1186/2251-6581-12-52

**Published:** 2013-12-19

**Authors:** Ahsana Shah, Mohammad Afzal

**Affiliations:** Human Genetics and Toxicology Laboratory, Section of Genetics, Department of Zoology, Aligarh Muslim University, Aligarh, Uttar Pradesh India

**Keywords:** Diabetes mellitus, Hypertension, Population, Manipur, India

## Abstract

**Background:**

Type 2 Diabetes mellitus (DM) and hypertension (HT) are among the most common non-communicable chronic diseases in developed and developing countries around the world. The study reports the prevalence of DM and HT and its influence from its possible risk factors.

**Methods:**

Individuals of both sexes (Male-1099, Female-669) belonging to six different populations were randomly selected and screened for diabetes and hypertension following from different districts of Manipur, which is a small hilly state, situated in the north eastern extreme corner of India sharing an international boundary with Myanmar (Burma). “Diabetes mellitus” and “hypertension” were defined by the American Diabetes Association and the Joint National Committee’s 7th Report guidelines, respectively.

**Results:**

The overall prevalence of diabetes and hypertension in the entire study population was found to be 16.63% and 18.16% respectively. About 13.8% individuals had shown co-prevalence of Diabetes Mellitus and Hypertension. The association of Diabetes Mellitus with different risk factors such as consumption of alcohol and difference in physical activities were found to be statistically significant. The association of Diabetes Mellitus with different populations and age groups are also statistically significant. The association between Hypertension with different populations and different physical activities were also found to be statistically significant.

**Electronic supplementary material:**

The online version of this article (doi:10.1186/2251-6581-12-52) contains supplementary material, which is available to authorized users.

## Introduction

Type 2 diabetes mellitus (DM) and Hypertension (HT) are among the most common chronic non-communicable diseases and multifactorial disorders affecting both developed and developing countries and occur at a higher prevalence in the older age group and result from both genetic and environmental etiological factors [1–3]. They are the main preventable risk factors for coronary heart disease, stroke, end-stage renal failure, disability and increased health-care costs. Although DM and HT are not among the top leading causes of death, such as cancer and stroke, these two diseases draw attention from the public due to their increasing trends, while cancer and stroke are declining [4]. Epidemiological and clinical studies have shown that these diseases often cluster in individuals and in families, and are collectively known as Syndrome X [5].

DM is a chronic disease increasing in explosive pattern in India [6]. The term DM describes a metabolic disorder of multiple etiologies characterized by chronic hyperglycemia with disturbances of carbohydrate, fat and protein metabolism resulting from defects in insulin secretion or insulin action or both. It continues to increase in numbers and significance, as changing lifestyles lead to reduced physical activity, and increased obesity [7–9]. DM is a disease of insidious onset and the symptoms, when they eventually appear, do not warrant immediate attention and thus remain undiagnosed at onset and even when diagnosed is often ignored by persons afflicted by it [10]. A strong genetic basis, environmental factors and lifestyle changes have been implicated in the etiology of type 2 DM [11]. The biggest increase in DM cases is expected in China and India. India currently, have around 40 million cases of DM and these numbers are projected to increase to 87 million by the year 2030 [12]. Anticipating an epidemic like increase in the number of diabetic patients India has been christened as the ‘diabetic capital of the world [13]. The prevalence of type 2 DM has risen from 1.2% to 11% over last three decades [14]. A patient who suffers from type 2 DM has a 2–4 times greater risk of death from cardiovascular causes than the patient without DM. The most common cause of death in the diabetic patient is heart disease. In addition, peripheral vascular disease, end-stage renal disease, blindness and amputations are common co-morbidities in diabetic patients [15].

Similarly, HT is considered to be one of the most common causes of morbidity and mortality affecting mankind. HT is a common health problem in developed countries and a major risk factor for cardiovascular diseases (CVD) [16]. HT exhibits an iceberg phenomenon where unknown morbidity exceeds the known morbidity. The prevalence of HT is rapidly increasing in developing countries and is said to be one of the leading causes of death and disability among the elderly [17]. Its prevalence is probably on the increase in developing countries where adoption of western lifestyles and the stress of urbanization both of which are expected to increase the morbidity associated with unhealthy lifestyles unfortunately are not on the decline [16]. Genetic and environmental factors are also reported to play a key role in HT, 90% of which are better classified as idiopathic. High blood pressure in adults has a high impact on the economy and on the quality of life of individuals with important implications for resource expenditures [18].

Several studies conducted in different ethnic groups show a close association between HT and DM, where the prevalence of HT is significantly higher in the patients with non insulin-dependent DM (NIDDM or type II DM). Both systolic and diastolic HT has been reported, and conclusive evidence indicates that the link between DM and essential HT is hyperinsulinemia [19]. The prevalence of HT is 1.5–2.0 times more in those with DM than in those without DM, whereas almost one-third of the patients with HT develop DM later [20].

The presence of hypertension in diabetic patients substantially increases the risks of coronary heart disease, stroke, nephropathy and retinopathy. When HT coexists with DM, the risk of CVD is increased by 75%, which further contributes to the overall morbidity and mortality of already high risk population. Generally HT in type 2 diabetic persons clusters with other CVD risk factors such as microalbuminuria, central obesity, insulin resistance, dyslipidaemia, hypercoagulation, increased inflammation and left ventricular hypertrophy [21]. This clustering risk factor in diabetic patients ultimately results in the development of CVD, which is the major cause of premature mortality in patients with type 2 DM. DM and HT are interrelated diseases that strongly predispose people to atherosclerotic cardiovascular disease, and hence have been referred to as “the bad companions” [22].

The objective of the present study is to determine the prevalence rate and risk factor associated with DM and HT in six different populations of Manipur as till date very little information exist about the prevalence of DM and HT from Manipur.

## Materials and methods

### Populations

Manipur is a small hilly state, situated in the north eastern extreme corner of India that connects the Indian subcontinent to South East Asia as a unique narrow passageway and shares an international boundary with Myanmar (Burma). It is bound by Nagaland in the north, Mizoram in the south, Assam in the west and Burma lies in the east. The survey was conducted in the given districts of Manipur i.e. Imphal East, Imphal West, Thoubal, Ukhrul and Senapati taking various populations viz. Manipur Muslims belonging to biradari Sheikh, Syed, Pathan and Mughal, Meitei (Hindu) and Naga.

Manipuri Muslims comprising 8.32% of the total population according to the 2001 census are mostly migrants who started coming to the state in the middle of the 16th century and they belong to Sheikh, Syed, Pathan or Mughal castes. They have been given different clan names which in Manipuri are called Yumnak or Sagei. About 74 clans are reported in Manipur in the present times [23–25]. Meitei are presumably formed by the admixture of Koomal, Looang, Moirang and Meitei, all of whom are reported to have arrived at different periods of time, coming from different directions and now represent the clans of the community [23]. While the Naga are the indigenous tribal population of Manipur, they belong to the Naga-Kuki-Chin group of the Tibeto-Burman linguistic family and are believed to have migrated to Manipur probably between 300 and 400 years ago from Burma [26].

A well designed, pre- tested questionnaire was used and a house to house survey was conducted and all the people above the age of 20 years were interviewed and examined. Informed written consent was obtained. Data on age, sex, occupation, marital status, personal habits were also collected.

Experimental research has been performed with the approval of an appropriate ethics committee and the research carried out on humans is compliance with the Helsinki Declaration. The work has been done under the approval of the Institutional Ethics committee headed by Dr Asad U Khan (Assoc. Professor & Coordinator), Interdisciplinary Biotechnology Unit, Aligarh Muslim University, Aligarh – 202002 (India), reference no. is Biot/2444.

Plasma FBS concentration was measured by finger stick with a glucometer. Recent studies have shown that modern handheld glucose measuring devices have excellent technical characteristics and yield results that are similar to the reference laboratory methods. Furthermore, various studies have reported that capillary glucose measurements are as suitable as venous glucose measurements in the diagnosis and detection of type 2 DM mellitus in epidemiological studies and may be cost effective in the implementation of pre-screening procedures [27]. Participants with levels ≥100 mg/dl subsequently underwent an OGTT2 to confirm the diagnosis of DM. DM was defined according to the ADA recommendations as FBS ≥126 mg/dl or oral glucose tolerance test OGTT2 ≥200 mg/dl [28]. Any resident who reported that he/she was a diabetic and on treatment was counted as a diabetic, irrespective of FBS values, and was not tested further.

Blood Pressure (BP) readings were within a gap of 15 minutes. BP was measured using a mercury sphygmomanometer by palpation and auscultation method in right arm in sitting position. Two readings were taken 15 min apart and the average of both the readings was taken for analysis. HT was also diagnosed based on anti HT medications or having a prescription of anti-hypertensive drugs and were classified as Hypertensive (HT) irrespective of their current BP reading or if the blood pressure was greater than 140/90 mmHg i,e systolic BP more than 140 and diastolic BP more than 90 mm of Hg – Joint National Committee 7 (JNC VII) Criteria [29]. About 294 and 321 individuals were suffering from diabetes and hypertension respectively. And around 244 individuals show the co prevalence of DM and HT. During the survey the individuals were enquired if they are under medication. The individuals who reported to be under medication only are counted as diabetic and hypertensive irrespective of their current FBS or BP reading.

Physical activity was divided into two categories viz non – active and active. The former includes elderly, retired executives, businessmen, housewives, professionals, teachers, skilled workers while the latter include farmers, service forces, manual labours and also those who performed any type of vigorous or moderate activity for at least 30 minutes a day for most of the days in a week other than routine daily activities.

Smokers in India consume tobacco in various forms: rolled tobacco leaves (bidi), Indian pipe (chillum, hookah), cigarettes and chewing tobacco. Anyone smoking in any form at least once a time per day for a minimum of past six months including ex-smokers was considered as smoker, and others were classified as non smokers [30]. Regarding consumption of alcohol, a “current drinker” was defined as that who consumed one or more drinks of any type of alcohol in the year preceding the survey [31]. Chi-square analysis using 2×2 contingency tables were used to compare any two groups and P < 0.05 was considered statistically significant.

## Results

### Prevalence of DM

**Figure 1:**
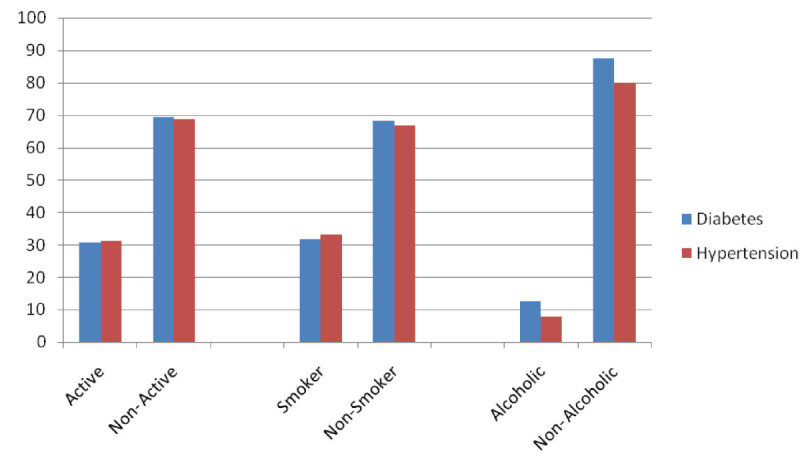
**Risk factors of diabetes and hypertension –activity, smoking and alcoholic intake.**

In total, 1768 individuals were taken for the present study. Out of this 294 (16.63%) individuals were found to be diabetic. Males show higher prevalence of DM i.e. 192 (17.47%) while 102 (15.25%) of females were diabetic. The effect of gender on causal of DM is found to be insignificant. Among six populations taken Sheikh shows the highest prevalence of DM i.e. 23.21% while the least DM prevalence was found in the Naga population, 10.17%. Individuals in different age groups shows variation in the prevalence of DM with highest prevalence occurring in the age group 40–60 (56.12%) followed by 60–80 (32.31%) age group and the least prevalence was shown by the individuals in the age group 20–40 (11.56%). The mean age of male and female suffering from DM is 55.68 and 49.90 respectively (Table 1). The presence of DM strongly correlated with physical activity in our present study i.e. 69.39% involved in non active physical activity showing cases of DM. Smoking and gender difference did not find any risk predictor of DM. The use of alcohol shows significant effect on the occurrence of DM with χ^2^-19.22, df-1, p-0.00. The chisquare test used to study the association between the prevalence of DM among different population (χ^2^-35.035, df-5, p-0.00) and different age group (χ^2^- 24.0178, df-10, p-0.0069) with consumption of alcohol and those with different physical activity and were found to be statistically significant (Tables 2, 3, 4 & 5, Figure 1). The results are also evident with the analysis of variance study (ANOVA) (Tables 6, 7 & 8).

**Table 1:** **Mean age of male and female and its association with age for the causal of diabetes and hypertension**

Age (yrs)	Diabetes		Hypertension	
	Male	Female	Male	Female
**20-30**	4	6	0	1
**30-40**	11	13	16	12
**40-50**	46	37	63	37
**50-60**	57	25	70	38
**60-70**	55	13	38	23
**70-80**	19	8	16	7
**Mean**	55.68	49.90	53.77	52.71
**SD**	11.58	12.65	10.57	10.89
**SE**	±0.84	±1.25	±0.74	±1.00
**t diff**	3.84	df-1	0.85	df-1
**p**	0.081		0.2757

**Table 2:** **Prevalence of diabetes among six different populations of Manipur**

Populations	Diabetes			Normal			Total		
	M	F	C	M	F	C	M	F	C
**Sheikh**	93 [25.20]	50 [20.24]	143 [23.21]	276 [74.80]	197 [79.76]	473 [76.79]	369	247	616
**Syed**	13 [11.61]	4 [8.00]	17 [10.49]	99 [88.39]	46 [92.00]	145 [89.51]	112	50	162
**Pathan**	28 [13.40]	14 [15.56]	42 [14.05]	181 [86.60]	76 [84.44]	257 [85.95]	209	90	299
**Moghul**	13 [18.57]	6 [18.18]	19 [18.45]	57 [81.43]	27 [81.82]	84 [81.55]	70	33	103
**Meitei**	23 [17.56]	20 [12.35]	43 [14.68]	108 [82.44]	142 [87.65]	250 [85.32]	131	162	293
**Naga**	22 [10.58]	8 [9.20]	30 [10.17]	186 [89.42]	79 [90.89]	265 [89.83]	208	87	295
**Total**	192 [17.47]	102 [15.25]	294 [16.63]	907 [82.53]	567 [84.75]	1474 [83.37]	1099	669	1768

Percentage shown in parentheses.

**Table 3:** **Association of populations with diabetes in Manipur**

	Sheikh	Syed	Pathan	Moghul	Meitei	Naga
**Diabetes**	143	17	42	19	43	30
	[102.43]	[26.94]	[49.72]	[17.13]	[48.72]	[49.06]
**Normal**	473	145	257	84	250	265
	[513.57]	[135.06]	[249.28]	[85.87]	[244.28]	[245.94]

The numbers represent the observed cases and the value in parentheses represents expected value. Χ^2^-35.035, df-5, p-0.00.

**Table 4:** **Association of sex with diabetes and hypertension in Manipur**

Disease	Male	Female		Disease	Male	Female	
**Diabetes**	192	102	Χ^2^-2.731	**Hypertension**	203	118	Χ^2^-0.1939
	(182.75)	(116.24)			(199.54)	(121.46)	
**Normal**	907	567	df-1	**Normal**	896	551	df-1
	(916.25)	(582.76)	p-0.098		(899.46)	(547.54)	p-0.6597

The numbers represent the observed cases and the value in parentheses represents expected value.

**Table 5:** **Association of age with diabetes in Manipur**

	A_1_(20–40)	A_2_(40–60)	A_3_(60–80)
**Sheikh**	7 (16.54)	90 (80.26)	46 (46.21)
**Syed**	2 (1.97)	12 (9.54)	3 (5.49)
**Pathan**	7 (4.86)	24 (23.57)	11 (13.57)
**Moghul**	1 (2.2)	7 (10.66)	11 (6.14)
**Meitei**	10 (4.97)	18 (24.13)	15 (13.89)
**Naga**	7 (3.47)	14 (16.89)	9 (9.69)

The numbers represent the observed cases and the value in parentheses represents expected value. Χ^2^-24.0178, df-10, p-0.0069.

**Table 6:** **Anova table for age, sex and disease in all populations**

Source	df	SS	MS	F
**Between all group**	2	15910.75	7955.4	
**Between Ages (A1,A2,A3)**	2	43784.75	21892.4	340.84*
**Between Sexes (B1,B2)**	1	96936.3	96936.3	1509.2*
**Between disease (C1,C2)**	1	60.75	60.75	0.946
**Within group**	11	706.5	64.23	

*Represents significant difference.

**Table 7:** **Anova table for population, life style and disease in all populations**

Source	df	SS	MSS	F
**Between all group**	5	21829.89	4365.98	
**Between Populations (A1,A2,A3,A4,A5,A6)**	5	21823.33	4364.67	569.8*
**Between life style (B1,B2,B3)**	3	18672.5	6224.17	812.55*
**Between disease (C1,C2)**	1	9.01	9.01	1.176
**Within group**	35	268	7.66	

*Represents significant difference.

**Table 8:** **Anova table for population, sex and disease in all populations**

Source	df	SS	MSS	F
**Between all group**	5	17431.25	3486.25	
**Between Populations (A1,A2,A3,A4,A5,A6)**	5	18904.17	3780.8	366*
**Between Sexes (B1,B2)**	2	14594.9	7297.45	706.4*
**Between disease (C1,C2)**	1	20.25	20.25	1.96
**Within group**	35	361.5	10.33	

*Represents significant difference.

### Prevalence of HT

In the present study, 321(18.16%) individuals were found to be hypertensive. Males again show higher prevalence of HT i.e. 203 (18.47%) against 118 (17.64%) in females. The effect of gender on the causal of HT is found to be insignificant. Out of the six populations taken in the present study Sheikh shows higher prevalence of HT i.e. 25.97% while the least prevalence of HT was found in the Meitei population i.e. 10.24%. Individuals in the age group of 40–60 (64.8%) show highest prevalence, followed by 60–80 (26.17%) age group and the least prevalence was shown by the individuals in the age group of 20–40 (9.03%). The mean age of male and female from HT is 53.77 and 52.71 respectively (Table 1). About 68.85% individuals, involved in non-active physical activity, show higher prevalence of HT. Smoking, alcohol use and gender difference did not find any risk predictor of HT. The chisquare test show the association between the prevalence of HT and difference in populations (χ^2^-52.1501, df-5, p-0.00) and also between HT and different type of physical activity to be statistically significant (Tables 4, 9, 10, & 11, Figure 1). The results are also evident with analysis of variance study (ANOVA) (Tables 6, 7 & 8).

**Table 9:** **Prevalence of hypertension among six different populations of Manipur**

Populations	Hypertension			Normal			Total		
	M	F	C	M	F	C	M	F	C
**Sheikh**	90 [24.39]	70 [28.34]	160 [25.97]	279 [75.61]	177 [71.66]	456 [74.03]	369	247	616
**Syed**	14 [12.50]	7 [14.00]	21 [12.96]	98 [87.5]	43 [86.00]	141 [87.04]	112	50	162
**Pathan**	36 [17.22]	17 [18.89]	53 [17.73]	173 [82.78]	73 [81.11]	246 [82.27]	209	90	299
**Moghul**	16 [22.86]	8 [24.24]	24 [23.30]	54 [77.14]	25 [75.76]	79 [76.70]	70	33	103
**Meitei**	19 [14.50]	11 [6.79]	30 [10.24]	112 [85.50]	151 [93.21]	263 [89.76]	131	162	293
**Naga**	28 [13.46]	5 [5.75]	33 [11.19]	180 [86.54]	82 [94.25]	262 [88.81]	208	87	295
**Total**	203 [18.47]	118 [17.64]	321 [18.16]	896 [81.53]	551 [82.36]	1447 [81.84]	1099	669	1768

Percentage shown in parentheses.

**Table 10:** **Association of populations with hypertension in Manipur**

Populations	Sheikh	Syed	Pathan	Moghul	Meitei	Naga
**Hypertension**	160	21	53	24	30	33
	[111.84]	[29.14]	[54.29]	[18.7]	[53.2]	[53.56]
**Normal**	456	141	246	79	263	262
	[504.16]	[132.59]	[244.71]	[84.3]	[239.80]	[241.44]

The numbers represent the observed cases and the value in parentheses represents expected value. Χ^2^-52.1501, df-5, p-0.00.

**Table 11:** **Association of age with hypertension in Manipur**

	A_1_(20–40)	A_2_(40–60)	A_3_(60–80)
**Sheikh**	11 (14.45)	106 (103.7)	43 (41.87)
**Syed**	2 (1.897)	14 (13.61)	5 (5.495)
**Pathan**	6 (4.788)	27 (34.34)	20 (13.87)
**Moghul**	3 (2.17)	16 (15.55)	5 (6.28)
**Meitei**	2 (2.71)	20 (19.44)	8 (7.85)
**Naga**	5 (2.98)	25 (21.38)	3 (8.64)

The numbers represent the observed cases and the value in parentheses represents expected value. Χ^2^-12.85512, df-10, p-0.2319.

### Co-prevalence of DM and HT

A total of 244 (13.8%) individuals had both DM and HT, with the highest prevalence in the population of Sheikhs i.e. 18.83% and the least was among Syeds i.e. 9.26%. Of the 294 individuals with DM, 139 (47.28%) had HT. Out of 321 individuals with HT, 105(32.7%) were reported with DM. The effect of risk factors on the co prevalence of DM and HT shows difference when compared with the occurrence of only DM or HT. Only alcohol consumption shows significant effect on co-occurrence of both the diseases, other factors do not (Table 12), when compared with the prevalence of diabetes and hypertension taken separately (Tables 13 & 14).

**Table 12:** **Effect of risk factors on co-prevalence of diabetes and hypertension**

	Sheikh	Syed	Pathan	Moghul	Meitei	Naga	
**Male**	73	11	26	11	17	24	Χ^2^-5.4979
**Female**	(77.02)	(9.96)	(25.89)	(10.62)	(19.25)	(19.25)	p-0.358,
	43	4	13	5	12	5	df-5
	(38.98)	(5.04)	(13.11)	(5.38)	(9.75)	(9.75)	
**(20–40)**	6	2	5	2	3	5	Χ^2^-7.219
**(40–60)**	(10.93)	(1.41)	(3.68)	(1.51)	(2.73)	(2.73)	p-0.705
**(60–80)**	76	10	21	9	16	17	df-10
	(70.84)	(9.16)	(23.82)	(9.77)	(17.71)	(17.71)	
	34	3	13	5	10	7	
	(34.23)	(4.43)	(11.51)	(4.72)	(8.56)	(24.14)	
**Active**	33	5	12	5	10	9	Χ^2^-0.5211
**Non-active**	(35.18)	(4.55)	(11.83)	(4.85)	(8.795)	(8.795)	p-0.99,
	83	10	27	11	19	20	df-5
	(80.82)	(10.45)	(27.17)	(11.15)	(20.20)	(20.20)	
**Smoker**	38	4	13	5	9	10	Χ^2^-0.5697
**No smoker**	(37.6)	(4.86)	(12.63)	(5.18)	(9.39)	(9.39)	p-0.989
	78	11	26	11	20	19	df-5
	(78.44)	(10.14)	(26.37)	(10.82)	(19.61)	(19.61)	
**Alcoholic**	1	0	0	0	12	20	Χ^2^-126.643
**Non-alcoholic**	(15.69)	(2.03)	(5.27)	(2.16)	(3.92)	(3.92)	P-0.00
	115	15	39	16	29	29	df-5
	(100.31)	(12.97)	(33.73)	(13.84)	(2.61)	(2.61)	

The numbers represent the observed cases and the value in parentheses represents expected value.

**Table 13:** **Effect of lifestyle on diabetes**

Habit	Diabetes	No diabetes	Odds ratio (OR)
**Non-active**	204	473	OR- 4.796,
**Active**	90	1001	p-0.00
**Smoker**	93	433	OR-1.1124
**Non-smoker**	201	1041	p-0.439
**Alcoholic**	37	82	OR-2.4440
**Non-alcoholic**	257	1392	p- 0.00

**Table 14:** **Effect of physical activity, smoking and alcohol on hypertension**

Habit	Hypertension	No hypertension	Odds ratio (OR)
**Non-active**	221	456	OR- 4.8029
**Active**	100	991	p-0.00
**Smoker**	106	420	OR- 1.2056
**Non-smoker**	215	1027	p-0.1569
**Alcoholic**	25	94	OR-1.2157
**Non-alcoholic**	296	1353	p-0.4039

### Analysis of variance study (ANOVA) Result

The analysis of variance study (ANOVA) Table 6 reveals that age and sex have significant effect on the disease among all the populations. There is no difference between the diseases for these factors. Similarly, Table 7 shows there is significant effect of populations on life style and the diseases. Again there is no difference of these factors between the diseases. The Table 8, reveals that there is significant effect of populations and sex on the prevalence of the diseases. Again there is no difference in between the diseases for these effects.

## Discussion

Our present study is the first attempt regarding the prevalence of DM and HT reported from Manipur. Till date, very little information exists about the prevalence of DM and HT from North-Eastern part of India which also includes Manipur.

Prevalence of DM in the present study is found to be 16.63%. The prevalence of DM in percentage in different states of India are as follows Maharashtra (39.8%), Delhi (32.5%), Tamil Nadu (40.3%), West Bengal (31.0%), Karnataka (34.5%) Andhra Pradesh (37.5%), Gujarat (28.9%) and Madhya Pradesh (33.7%) [32]. It also revealed that the prevalence in the southern part of India is higher i.e. 13.5% Chennai, 12.4% Bangalore and 16.6% Hyderabad compared to Eastern India (Kolkatta) 11.7%, Northern India (New Delhi) 11.6% and Western India (Mumbai) 9.3% [33]. Survey in different countries are also present viz. Mexican Americans 25.7%, aboriginal Australians 25%, non Hispanic blacks 19.8% has been reported. Sheikh population show the highest prevalence i.e. 23.21% [34]. The association between prevalence of DM among those with different population is found to be statistically significant. Overall in the present study, males shows higher prevalence in comparison to females but the difference was not statistically significant. Prevalence percentage of DM among males is 17.47% while that of females is found to be 15.25%. Similar study in karnataka reports 18.8% of males and 14.4% of females to be diabetic [35]. Similar findings were reported by various researchers in India regarding female preponderance in Indian diabetics especially by Venkatesham et al. and Ramachandran et al. observed male excess amongst the diabetic person’s [36, 37]. A study conducted in Turkey has found high prevalence of type-2 DM among women [38].

Overall prevalence of HT in Manipur is found to be 18.16%. HT prevalence was found to be higher in comparison to DM in all the populations taken in the present study except for Meiteis. It is revealed that the prevalence percentage in Maharashtra is (56.4%), Delhi (48.2%), Tamil Nadu (39.5%), West Bengal (46.5%), Karnataka (32.1%), Andhra Pradesh (49.4%), Gujarat (45.3%) and Madhya Pradesh (52.0%) (32). The prevalence of HT has been increasing in India, both in rural and urban regions. Prevalence rate of HT was found to be 25.2 percent in a study of rural population in Tamil Nadu. The prevalence of HT in the urban population of West Bengal, representing eastern India, was reported to be 24.9%. The prevalence of HT among the urban population of Trivandrum city in Kerala in the south western India was reported to be 33.5% in the age groups between 45 and 64 years [39, 40]. Sheikh again shows higher prevalence of HT i.e. 25.97%. The association between prevalence of HT among those with different population is also found to be statistically significant. In present study overall males shows higher i.e. 18.47% than females which shows prevalence percentage of 17.64% but the difference was not statistically significant. This could be possibly because of the increased prevalence of risk factors of HT in males. Gupta et al. and Guang Hui Dong et al. also found it more in males [41, 42]. But Hazarika NC et al. reports lower prevalence of HT among males [43].

Present study shows the following order of prevalence of DM in different age groups viz (40–60) > (60–80) > (20–40). Age group of 40–60 shows prevalence percentage of 56.12%. Chisquare test shows age is significantly associated with DM prevalence. Most of the diabetics were in the age group of 50–60 years study in a conducted at rural Wardha [44]. A different study reported in elderly population (age ≥ 65 years) prevalence of type 2 DM increases with the increasing age but different findings were observed in the present study i.e. type 2 DM are more common at age between (40–60), like one reported by Aditya etal which tells that type 2 DM are more risky mainly at 51–60 years of age. Some other studies have reported in Indian population show the onset of T2DM in different age groups compared with Europeans. Age probably represents accumulation of environmental influences and the effect of genetically programmed senescence in the body systems [45–47].

Again the present study shows the following order of prevalence of HT in different age groups viz (40–60) > (60–80) > (20–40). Most of the studies agree with the fact that prevalence of HT increased with age. But in Manipur a slightly different observation could be seen. Since highest number of DM and HT was found in the age group of (40–60) years. This could be due to less number individuals suffering from diabetes and HT to survive. Chisquare test shows age is not significantly associated with the HT prevalence. The prevalence of HT in rural areas of Tamil Nadu in the age group of 45 – 60 years was 33% [48]. While Gupta et al. reported a prevalence of 24% in males and 17% in females in the age group of 20 years and above from rural Rajasthan [41]. Gilberts et al. carried out a study in rural Tamil Nadu in the age group of 20 years and above and found a prevalence of 12.5% [49]. From eastern India, Hazarika et al. reported a prevalence of 33.3% in the age group of 30 years and above, among the native populations of Assam [43]. Guang Hui Dong et al. from rural China reported a prevalence of 37.8% in the age group of 35 years and above. Difference in prevalence of various studies observed may be due to the variation in age group, geographical differences and diagnostic criteria adopted by authors [42].

Recent years have witnessed a rapid rise in the prevalence of DM and HT in India. This could be attributed to the increasing levels of sedentarism, urbanization, and consumption of energy-dense but fiber and micronutrient deficient food [50]. Additionally, Indians have a high intake of common salt. Manipuri people consumed rice as only cereal, and follow a non vegetarian diet, used chilies, both green and red, to make the food hot but a less-fatty dietary pattern. Manipuri people are known to have high physical activity. Mitra et al. [51] reported that Type 2 DM prevalence rates are higher in the east coast of Andhra Pradesh, particularly in Eluru and Tenali where rice is traditionally consumed as only cereal. The prevalence rate in urban and rural Hyderabad is low because of the presence of mainly wheat eating communities. One of the main reasons for proliferating diabetic cases in the state is the intake of rice as only cereal, sedate lifestyle, in take of junk food, obesity and inactive routine. The Naga tribes in the present study were found to be less suffering from DM and HT though they also take rice, and non-vegetarian diets, like the other five population taken in the present study. Besides Alcohol consumption and smoking was also found to be very high among them. The probable reason is that since the Naga population were mostly inhabitants of hilly areas, thier physical activity is very active since for daily chores like fetching water, marketing and going to work place they need to move up and down the hills and they travel long distances by foot, since used of vehicles is not possible in most of the cases to climb the hills. While the other population studied are mostly inhabitants of valley areas, where all modern facilities are available thus leading to less physical activities.

Alcohol was found to be positively associated with DM in this study. An association between alcohol and DM has been documented by many authors. There is a non-linear relationship between alcohol intake and the risk of type 2 DM [52]. Levitt et al., on the other hand, reported that alcohol intake was not a significant risk factor for DM in South Africa [53]. Smoking was not found to be positively associated with DM in this study. Vashitha etal, in study of rural population of Haryana [45] reported Smoking and alcohol use were not found to be a risk predictor of diabetes mellitus. Neither smoking nor alcohol intake was found to be positively associated with HT in the present study. Only physical activity shows positive association with both DM and HT.

Several studies show close association between HT and DM. In a study in France, Marre et al. reported HT in almost one-third of diabetic cases [54]. Among adult Afro-Americans, elevation of blood pressure is significantly higher in individuals with impaired glucose tolerance and DM than in nondiabetics. The occurrence of HT in diabetic patients increases significantly the risk of coronary artery disease, mortality and nephropathy. It is of significance that BP is controlled in diabetic patients. High blood pressure in diabetics hints at syndrome X or the metabolic syndrome which includes HT, hyperglycemia, obesity and hyperlipidemia. Physiological maneuvers, such as calorie and salt restriction and regular physical exercise, are shown to improve tissue sensitivity to insulin and hence lower blood pressure both in normotensive and hypertensive diabetics. Hence, control of DM and HT by appropriate methods, particularly dietary restriction of calories and sodium, and regular physical exercise, must be highlighted in order to decrease both the prevalence of HT and NIDDM [55]. Today, DM and HT no longer remain a disease of the high socioeconomic status or confined to urban area. However, not enough prevalence studies have been conducted in India for rural population. American Diabetes Association has proposed the screening of all the patients aged over 45 years by measuring fasting blood glucose, every three years, in addition to screening patients from high-risk groups and younger patients with HT, obesity, the family history of DM in the first-degree relative, and a family history of gestational DM.

The results of our present screening are only the representative study of six different populations of the state, in which the steady rise in DM and HT is observed. The need of the hour, therefore is that large scale epidemiological studies for other populations be undertaken to ascertain the causes of the rising Type 2 DM and HT epidemic in the state of Manipur, either for reducing or if possible, some serious efforts must be taken viz. like concerted preventive measures need to taken by the Government, health planners by discussing it with the general public steps to promote physical activity, changing societal perceptions of health and improving knowledge about their associated risk factors like hyperlipidemia, microalbuminuria and central obesity. Moreover, detection of the progenitors—prediabetes and prehypertension—through periodic surveillance can suggest forearly intervention and delay of disease progression [32]. Of interest is the prospect of introduction of presymptomatic diagnosis of susceptible individuals using genetic markers, where it is expected to play an essential role in the control and prevention of noncommunicable diseases. If this becomes a practical approach, the prevention of the complications will be of appreciable benefit to those prone to DM and HT [55]. It remains for the health planners to obtain the area specific information in order to assess the extent of the problem and identify areas for intervention of the disease to chalk out effective plan for the future.

## Conclusions

Large scale epidemiological studies for other populations needs to be undertaken to ascertain the causes of the rising Diabetes Mellitus and Hypertension epidemic in the state of Manipur, initiative for reducing, or if possible serious efforts must be taken by the Government and health planners to prevent the rising burden of Diabetes Mellitus and Hypertension on the state.

## Authors’ original submitted files for images

Below are the links to the authors’ original submitted files for images. Authors’ original file for figure 1
