# Supplementary figures and images for: Prevalence of diabetes and hypertension and association with various risk factors among different Muslim populations of Manipur, India
Source: J Diabetes Metab Disord. 2013 Dec 19;12:52. doi: 10.1186/2251-6581-12-52 (PMC7968346; doi:10.1186/2251-6581-12-52)

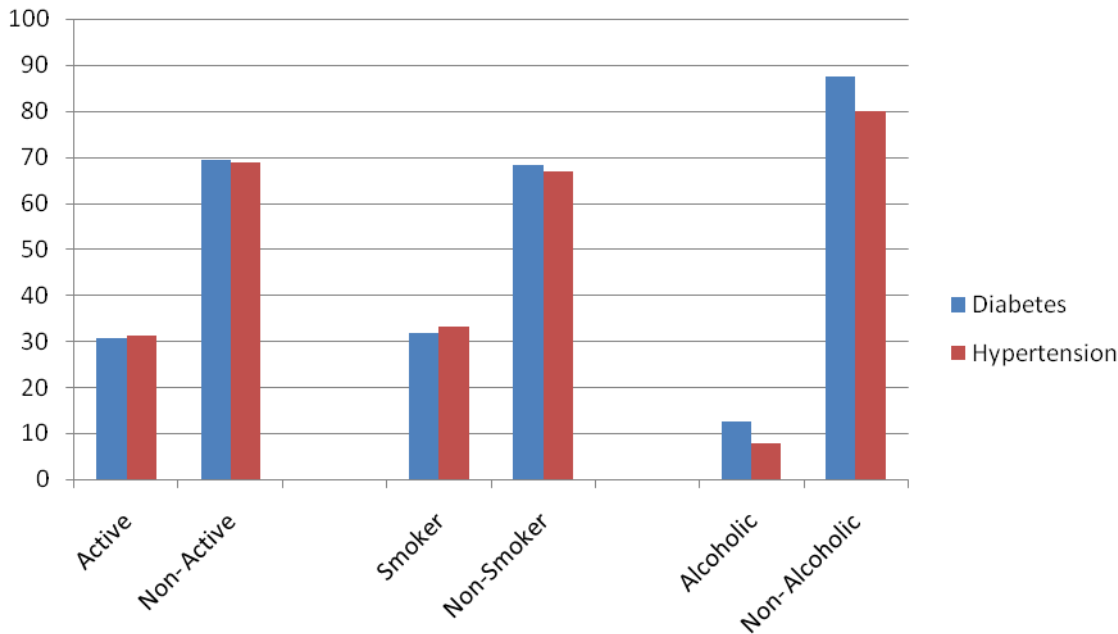

Supplement: Supplementary file 1 — Authors’ original file for figure 1 [file 40200_2013_184_MOESM1_ESM.pdf]
